# Walking and Mortality in Japan: The Miyagi Cohort Study

**DOI:** 10.2188/jea.14.S26

**Published:** 2005-03-18

**Authors:** Kazuki Fujita, Hideko Takahashi, Chihaya Miura, Takayoshi Ohkubo, Yuki Sato, Takashi Ugajin, Kayoko Kurashima, Yoshitaka Tsubono, Ichiro Tsuji, Akira Fukao, Shigeru Hisamichi

**Affiliations:** 1 Division of Epidemiology, Department of Public Health and Forensic Medicine, Tohoku University Graduate School of Medicine.; 2 Department of Public Health, Yamagata University School of Medicine.

**Keywords:** all-cause mortality, prospective cohort study, time spent walking,

## Abstract

BACKGROUND: Although many studies in western populations demonstrated that time spent walking was associated with a reduced risk of all-cause mortality, data on Japanese has been sparse.

METHODS: In 1990, 20,004 men and 21,159 women in Miyagi Prefecture in rural northern Japan (40-64 year of age) completed a self-administered questionnaire including a question on time spent walking. Cox regression was used to estimate relative risk (RR) of mortality according to three levels of walking (30 minutes or less, between 30 minutes and one hour, and one hour or more), with adjustment for age, education, marital status, past history of diseases, smoking, drinking, body mass index, and dietary variables. During 11 years of follow-up, 1,879 subjects had died.

RESULTS: Time spent walking was inversely associated with risk of all-cause mortality: compared with men and women who walked one hour or more per day, multivariate RR (95% confidence intervals) was 1.06 (0.95-1.19) for subjects who walked between 30 minutes and one hour per day, and 1.16 (1.04-1.29) for subjects who walked 30 minutes or less per day (P for trend=0.007). Shorter duration of walking was associated with increased mortality among men who were never smokers (P for trend=0.081) and past smokers (P for trend=0.026), but not among currently smoking men (P for trend=0.751). We observed similar effect modification for women.

CONCLUSIONS: Time spent walking was associated with a reduced risk for all-cause mortality, especially among nonsmoking men and women.

Epidemiologic evidence demonstrated that physical activity was associated with a reduced risk of all-cause mortality.^1^^-^^4^ Walking, one of the major moderate-intensity physical activities, has been a widely accepted means for promoting the adoption of physically active lifestyles among adults across the all ages. Previous epidemiologic studies, therefore, used the time spent walking,^5^^-^^12^ as well as walking pace^12^^,^^13^ or distance walked,^14^^,^^15^ as an indicator of physical activity.

Among middle-aged and older adults, time spent walking was associated with a reduced risk of all-cause mortality in western populations.^6^^-^^9^ However, few evidence exist for the Japanese population. Two studies in Japan demonstrated an inverse relation between time spent walking and all-cause mortality among men and women aged 40 years and older.^10^^,^^11^ However, the two studies used relatively small numbers of subjects so that they did not observe a statistically significant association after excluding subjects who had died during the first one or two years of follow-up.

The purpose of this study was to examine the relation between time spent walking and all-cause mortality in a large Japanese population-based prospective cohort study, using a validated self-administered questionnaire on walking.

## METHODS

### Study Cohort

We have reported the design of this prospective cohort study in detail elsewhere.^16^ Briefly, from June through August 1990, we delivered a self-administered questionnaire on various health habits to 51,921 subjects (25,279 men and 26,642 women) who were 40-64 years of age and lived in 14 municipalities of Miyagi Prefecture in northern Japan. The questionnaires were delivered to and collected from the subjects’ residences by members of health promotion committees appointed by the municipal governments. Usable questionnaires were returned from 47,605 subjects (22,836 men and 24,769 women), yielding a response rate of 91.7%.

The study protocol was approved by the institutional review board of Tohoku University Graduate School of Medicine. We considered the return of the self-administered questionnaires signed by the subjects to imply their consent to participate in the study.

### Exposure Data

For the assessment of time spent walking, the questionnaire asked “How long do you walk a day on average?” and the subjects were asked to choose one out of three options; 30 minutes or less, between 30 minutes and one hour, and one hour or more.

We conducted a validation study for the questionnaire assessment of time spent walking.^17^ Specifically, 106 subjects (51 men and 55 women, mean 61.7 years) in the study distinct completed the questionnaire five times at 3-month intervals. Along with the first through the fourth questionnaire surveys, pedometer measurement was conducted for three consecutive days. The sex- and age-adjusted mean daily numbers of walking steps counted by the pedometer were 5,857, 7,047, and 7,621 for the three categories of walking questions in the fifth questionnaire, and it showed significant linear associations with all of the five questionnaire measurements.

### Follow-up

Of 47,605 subjects who responded to the questionnaire, we excluded 1,522 subjects who indicated that they had prior histories of cancer (n=561), stroke (n=379), or myocardial infarction (n=582). We also excluded 539 subjects who had prevalent cancer, which we ascertained by record linkage to the population-based cancer registry covering the study area.^16^ We further excluded subjects who had incomplete responses for walking question (n=4,381). Consequently, 41,163 subjects (20,004 men and 21,159 women) with 1,879 deaths (1,255 men and 624 women) were included in this analysis.

We followed up vital and residential status of subjects from June 1, 1990, through March 31, 2001. For this follow-up, we established the Follow-up Committee that was consisted of Miyagi Cancer Society; Community Health Division of all 14 municipalities; Department of Health and Welfare, Miyagi Prefectural Government; and Division of Epidemiology, Tohoku University Graduate School of Medicine. The Committee periodically reviewed the Residential Registration Record of each municipality. With this review, we identified the subjects who either died or emigrated during observation. For both decedents and emigrants, we recorded the date of death or emigration. For decedents, we investigated cause of death by reviewing the death certificates of the subjects at Public Health Centers of the study area. The underlying cause of death was coded according to International Classification of Diseases, the Ninth Revision (ICD9). We discontinued follow-up of subjects who emigrated from the study municipalities because of logistical limitations.

We counted person-years of follow-up for each subject from June 1, 1990, until the date of death, date of emigration outside the study districts, or the end of the study period (March 31, 2001), whichever occurred first. A total of 426,305 person-years accrued. There were 1,991 subjects (4.8 % of the analytic cohort) who emigrated from the study municipalities and were lost to follow-up.

### Statistical Analysis

We used Cox proportional-hazards regression to estimate relative risk (RR) and 95% confidence interval (CI) of all-cause mortality according to categories of time spent walking per day and to adjust for potentially confounding variables, using the PHREG procedure on SAS^®^ version 8.2 statistical software package (SAS Inc., Cary, NC, USA).

We considered the following variables as potential confounders: age in years; education (up to 15 years of age, 16-18, or 19 years or older); marital status at baseline (whether or not living with spouses); past histories of hypertension, renal diseases, liver diseases, diabetes mellitus, peptic ulcers, or tuberculosis; cigarette smoking (never smokers, past smokers, current smokers smoking 1-19 cigarettes per day, or current smokers smoking at least 20 cigarettes per day); alcohol drinking (never drinkers, past drinkers, or current drinkers); body mass index in kg/m^2^ (less than 18.5, 18.5-24.9, or 25.0 or higher); and consumption frequencies of green vegetables and oranges (almost daily, 3-4 times per week, 1-2 times per week, or 1-2 times per month or less often).

We repeated all analyses after excluding the subjects who died during the first three years of follow-up. We also conducted stratified analyses according to the categories of covariates included in the multivariate analyses to examine whether the association between time spent walking per day and all-cause mortality was modified by these variables. P values for tests of linear trends were estimated by treating the three categories of time spent walking as an ordinal variable. All P values were two-tailed.

## RESULTS

Table 1 outlines the characteristics of the study population by levels of time spent walking per day. The proportions of men who walked 1 hour or more per day (the highest category), those who walked between 30 minutes and one hour per day (the middle category), and those who walked 30 minutes or less per day (the lowest category) were 45.6%, 23.6%, and 30.8%, respectively. Corresponding proportions among women were 45.7%, 24.8%, and 29.5%, respectively. Compared with men who walked 30 minutes or less per day, those who walked one hour or more per day were older, less likely to be obese, and more likely to consume green vegetables and oranges daily. Compared with women who walked 30 minutes or less per day, those who walked one hour or more per day were older and more likely to consume green vegetables daily.

**Table 1.** Baseline characteristics of subjects by time spent walking per day.

Characteristics	Men			Women		
	
	Time Spent Walking per Day (hr)			Time Spent Walking per Day (hr)		
	
	≥1	1.0-0.5	≤0.5	≥1	1.0-0.5	< 0.5
No. of subjects	9,130	4,720	6,154	9,676	5,238	6,245
Mean age ( SD )	52.3 (7.5)	51.1 (7.7)	50.0 (7.5)	52.6 (7.2)	52.1 (7.5)	50.4 (7.5)
Body mass index (%)						
<18.5	2.0	1.9	2.2	2.6	2.5	3.0
18.5-24.9	72.7	69.5	67.9	67.0	65.3	65.9
25.0≤	25.3	28.6	29.9	30.4	32.2	31.1
Education, age (%)						
<15	46.0	34.4	35.0	41.4	36.9	36.8
16-18	43.2	47.1	47.7	46.9	48.6	49.0
19≤	10.8	18.5	17.3	11.7	14.5	14.2
Living with spouse (%)						
Yes	92.8	92.8	92.2	88.0	86.4	87.7
No	7.2	7.2	7.8	12.0	13.6	12.3
Past history (%)						
Hypertention	16.9	19.2	18.0	19.2	21.5	19.0
Renal diseases	2.7	3.2	3.6	3.4	4.0	4.1
Liver diseases	5.9	7.1	6.6	3.4	3.3	3.8
Diabetes mellitus	4.4	6.1	5.6	2.5	3.5	3.4
Peptic ulcers	19.0	21.5	21.6	9.0	9.2	10.2
Tuberculosis	2.5	3.8	3.4	2.0	2.6	2.8
Green vegetables (%)						
≤1-2 times/month	13.5	14.2	17.9	8.5	7.1	9.7
1-2 times/week	32.7	36.5	38.1	27.1	29.8	32.7
3-4 times/week	30.8	30.0	27.9	35.1	36.7	35.1
Everyday	22.9	19.3	16.0	29.3	26.4	22.6
Oranges (%)						
≤1-2 times/month	28.9	27.6	32.0	16.5	13.7	16.0
1-2 times/week	27.7	32.2	31.5	20.5	21.2	20.9
3-4 times/week	23.8	24.0	20.9	26.5	27.7	27.6
Everyday	19.7	16.3	15.5	36.5	37.5	35.5
Smoking status (%)						
Never	20.0	19.4	18.7	90.0	90.4	88.3
Past	18.9	22.2	19.8	2.0	1.7	2.4
Current	61.1	58.4	61.5	8.0	7.9	9.3
Alcohol drinking (%)						
Never	15.5	14.2	16.3	71.4	70.6	67.9
Past	6.5	6.4	7.0	3.6	3.8	4.3
Current	78.0	79.4	76.7	25.0	25.6	27.8

SD: standard deviation

Table 2 presents RRs for all-cause mortality according to categories of time spent walking per day. Age- and sex-adjusted analysis showed that there was a significant, dose-response inverse relation between time spent walking per day and all-cause mortality (P for trend<0.001). The risk of death among the lowest category increased by 22% compared with the highest category. These results remained basically unchanged after adjustment for potential confounders and after the exclusion of death occurring in the first 3 years of follow-up.

**Table 2.** Relative risk (RR) and 95% confidence interval (CI) of all-cause mortality by time spent walking.^†^

	Time Spent Walking (hour per day)			P for trend

	≥1	1.0 - 0.5	≤0.5	
Person-years	196,079	102,779	127,448	
No. of death	858	455	566	
Age and sex-adjusted RR	1.00	1.09 (0.95 - 1.22)	1.22 (1.09 - 1.35)	<0.001
Multivariate RR1	1.00	1.06 (0.95 - 1.19)	1.16 (1.04 - 1.29)	0.007
Multivariate RR2	1.00	1.06 (0.93 - 1.20)	1.17 (1.04 - 1.31)	0.011

† : Adjusted for age in years; sex; education (up to 15 years of age, 16-18, or 19 years or older); marital status at baseline (whether or not living with spouse); past histories of hypertension, renal diseases, liver diseases, diabetes mellitus, peptic ulcers, or tuberculosis; cigarette smoking (never smokers, past smokers, current smokers smoking 1-19 cigarettes per day, or current smokers smoking at least 20 cigarettes per day); alcohol drinking (never drinkers, past drinkers, or current drinkers); body mass index in kg/m^2^ (less than 18.5, 18.5-24.9, or 25.0 or higher); consumption frequencies of green vegetables and oranges (almost daily, 3-4 times per week, 1-2 times per week, or 1-2 times per month or less often). RR2 has been estimated with the exclusion of subjects who died within the first 3 years of follow-up. Numbers in parentheses are 95% confidence intervals.

Table 3 presents the results of separate analyses for men and women. Among men, age-adjusted RR was higher among the lowest category compared with the highest category, although this result was attenuated after adjustment for potential confounders. Among women, age-adjusted analysis showed that there was a significant inverse relation between time spent walking and all-cause mortality (P for trend<0.001). The risk of death among the lowest category increased by 40% compared with the highest category. The risk remained significantly increased after adjustment for potential confounders and after the exclusion of death occurring in the first 3 years of follow-up.

**Table 3.** Relative risk (RR) and 95% confidence interval (CI) of all-cause mortality by time spent walking for men and women.^†^

Variable	Time Spent Walking (hour per day)			P for trend

	≥1	1.0 - 0.5	≤0.5	
Men				
Person-years	94,600	48,307	62,774	
No. of death	598	288	369	
Age-adjusted RR	1.00	1.03 (0.90 - 1.19)	1.14 (1.00 - 1.30)	0.061
Multivariate RR1	1.00	1.00 (0.87 - 1.15)	1.10 (0.96 - 1.25)	0.205
Multivariate RR2	1.00	0.98 (0.84-1.14)	1.08 (0.94 - 1.25)	0.318

Women				
Person-years	101,479	54,472	64,673	
No. of death	260	167	197	
Age-adjusted RR	1.00	1.23 (1.01 - 1.49)	1.40 (1.16 - 1.68)	<0.001
Multivariate RR1	1.00	1.21 (0.99 - 1.47)	1.34 (1.11 - 1.62)	0.002
Multivariate RR2	1.00	1.24 (1.00 - 1.54)	1.38 (1.12 - 1.70)	<0.001

†: Adjusted for age in years; education (up to 15 years of age, 16-18, or 19 years or older); marital status at baseline (whether or not living with spouse); past histories of hypertension, renal diseases, liver diseases, diabetes mellitus, peptic ulcers, or tuberculosis; cigarette smoking (never smokers, past smokers, current smokers smoking 1-19 cigarettes per day, or current smokers smoking at least 20 cigarettes per day); alcohol drinking (never drinkers, past drinkers, or current drinkers); body mass index in kg/m^2^ (less than 18.5, 18.5-24.9, or 25.0 or higher); consumption frequencies of green vegetables and oranges (almost daily, 3-4 times per week, 1-2 times per week, or 1-2 times per month or less often). RR2 has been estimated with the exclusion of subjects who died within the first 3 years of follow-up. Numbers in parentheses are 95% confidence intervals.

Table 4 presents the results of stratified analysis according to smoking status among men. Among never smokers, those who walked 30 minutes or less per day had an increased risk of mortality, compared with those walked one hour or more per day (P for trend=0.04). After adjustment for potential confounders, men in the lowest category had an RR of 1.42 (95% CI, 0.97-2.06), compared with men in the highest walk category, although P-value for trend was marginally significant (P for trend=0.081). Among past smokers, there was a significant inverse association between time spent walking and mortality (P for trend=0.026), whereas, among current smokers, we did not observe such association (P for trend=0.751).

**Table 4.** Relative risk (RR) and 95% confidence interval (CI) of all-cause mortality by time spent walking for men according to smoking status.^†^

	Time Spent Walking (hour per day)			P for trend

	≥1	1.0 - 0.5	≤0.5	
Never smoker				
Person-years	17,959	9,082	11,294	
No. of death	65	28	52	
Age-adjusted RR	1.00	1.00 (0.64 - 1.56)	1.49 (1.03 - 2.15)	0.041
Multivariate RR1	1.00	1.00 (0.64 - 1.56)	1.42 (0.97 - 2.06)	0.081
Multivariate RR2	1.00	1.03 (0.64 - 1.68)	1.47 (0.98 - 2.21)	0.071

Past smoker				
Person-years	16,870	10,231	11,866	
No. of death	88	65	77	
Age-adjusted RR	1.00	1.31 (0.95 - 1.81)	1.50 (1.10 - 2.04)	0.008
Multivariate RR1	1.00	1.27 (0.92 - 1.76)	1.42 (1.04 - 1.94)	0.026
Multivariate RR2	1.00	1.37 (0.96 - 1.96)	1.40 (0.98 - 1.98)	0.054

Current smoker				
Person-years	57,681	28,307	38,738	
No. of death	429	191	234	
Age-adjusted RR	1.00	0.99 (0.83 - 1.17)	1.02 (0.87 - 1.20)	0.852
Multivariate RR1	1.00	0.95 (0.80 - 1.13)	0.98 (0.83 - 1.15)	0.751
Multivariate RR2	1.00	0.91 (0.75 - 1.10)	0.97 (0.81 - 1.15)	0.607

† : Adjusted for age in years; education (up to 15 years of age, 16-18, or 19 years or older); marital status at baseline (whether or not living with spouse); past histories of hypertension, renal diseases, liver diseases, diabetes mellitus, peptic ulcers, or tuberculosis; alcohol drinking (never drinkers, past drinkers, or current drinkers); body mass index in kg/m^2^ (less than 18.5, 18.5-24.9, or 25.0 or higher); consumption frequencies of green vegetables and oranges (almost daily, 3-4 times per week, 1-2 times per week, or 1-2 times per month or less often). RR2 has been estimated with the exclusion of subjects who died within the first 3 years of follow-up. Numbers in parentheses are 95% confidence intervals.

We conducted similar analyses among women. Among never smokers, there was a significant, inverse dose-response relation between time spent walking and mortality, (for women in the moderate category, RR=1.35, 95% CI=1.03-1.78; for women in the lowest category, RR=1.65, 95% CI=1.28-2.14; P for trend<0.001). Among current smokers, there was no relation between time spent walking and mortality: the RR for women in the moderate and the lowest categories were 0.46 (95% CI, 0.19-1.16) and 1.01 (95% CI, 0.53-1.93), respectively (P for trend=0.93). A small number of past smokers (331 subjects with 13 deaths) prohibited informative analysis.

Although we conducted stratified analyses according to covariates other than smoking status, we did not detect substantial modification of the association between time spent walking and all-cause mortality (data not shown).

## DISCUSSION

In this prospective cohort study of middle-aged men and women in rural Japan, there was a significant dose-response inverse relation between time spent walking per day and the risk of all-cause mortality in the total population. We also observed that the association between time spent walking and all-cause mortality was modified by smoking status: shorter time of walking was significantly associated with an increased risk of mortality in nonsmokers, while the association was not apparent in current smokers.

Previous studies have shown an inverse relation between time spent walking and all-cause mortality in both sexes combined,^9^^,^^10^ men,^7^^,^^8^^,^^11^ and women.^6^^,^^7^^,^^11^ In Japanese population, however, only two studies were conducted to examine this relationship. Seki et al.^10^ demonstrated that the risk of all-cause mortality among men and women who walked more than 1 hour per day decreased by 26% compared with those walked less than 1 hour per day. Morioka et al.^11^ showed that the risk of all-cause mortality among women who walked 30 minutes or less per day increased by 81% compared with those walked more than 30 minutes per day, although this strong association was not observed in men.

In the present study, we found a significant inverse association between time spent walking and mortality among women, but not among men. This gender difference would be attributed to the difference in proportion of never-smokers between men and women (18.8% in men, 89.0% in women), since we observed the inverse association among men and women who never smoked. Thus, our findings support the consistent inverse association between time spent walking and mortality among never-smoking middle-aged men and women.

Strengths of our study include prospective cohort design with a large sample size. The size of our study, which included 20,004 men and 21,159 women, of whom 1,255 men and 624 women died, was much larger than those in other reports^6^^,^^8^^,^^10^^,^^11^ and was sufficient to detect the associations. Furthermore, the distribution of the causes of death among the study subjects was consistent with the national average.^18^ Other strengths of our study include the use of the reproducible and valid questionnaire^17^ and adjustment for a large number of potential confounders.

Our study had some limitations. We did not measure other variables related to walking such as walking pace, partly because we used the single-item questionnaire on time spent walking. Since it is well known that walking pace is higher among the middle-aged men compared with the middle-aged women,^19^ the impact of time spent walking on mortality among men might be underestimated.

In conclusion, this population-based prospective cohort study in rural Japan showed that time spent walking was associated with a reduced risk for all-cause mortality, especially among nonsmoking men and women.
